# *Dehalococcoides* as a Potential Biomarker Evidence for Uncharacterized Organohalides in Environmental Samples

**DOI:** 10.3389/fmicb.2017.01677

**Published:** 2017-09-01

**Authors:** Qihong Lu, Ling Yu, Zhiwei Liang, Qingyun Yan, Zhili He, Tiangang Luan, Dawei Liang, Shanquan Wang

**Affiliations:** ^1^Environmental Microbiome Research Center and the School of Environmental Science and Engineering, Sun Yat-sen University Guangzhou, China; ^2^State Key Laboratory of Pest Control and Resource Utilization, School of Life Sciences, Sun Yat-sen University Guangzhou, China; ^3^Beijing Key Laboratory of Bio-inspired Energy Materials and Devices, School of Chemistry and Environment, Beihang University Beijing, China; ^4^Guangdong Provincial Key Laboratory of Environmental Pollution Control and Remediation Technology Guangzhou, China

**Keywords:** *Dehalococcoides*, biomarker, environmental samples, organohalide compounds, reductive dehalogenation

## Abstract

The massive production and improper disposal of organohalides resulted in worldwide contamination in soil and water. However, their environmental survey based on chromatographic methods was hindered by challenges in testing the extremely wide variety of organohalides. *Dehalococcoides* as obligate organohalide-respiring bacteria exclusively use organohalides as electron acceptors to support their growth, of which the presence could be coupled with organohalides and, therefore, could be employed as a biomarker of the organohalide pollution. In this study, *Dehalococcoides* was screened in various samples of bioreactors and subsurface environments, showing the wide distribution of *Dehalococcoides* in sludge and sediment. Further laboratory cultivation confirmed the dechlorination activities of those *Dehalococcoides*. Among those samples, *Dehalococcoides* accounting for 1.8% of the total microbial community was found in an anaerobic granular sludge sample collected from a full-scale bioreactor treating petroleum wastewater. Experimental evidence suggested that the influent wastewater in the bioreactor contained bromomethane which support the growth of *Dehalococcoides*. This study demonstrated that *Dehalococcoides* could be employed as a promising biomarker to test the present of organohalides in wastestreams or other environmental samples.

## Introduction

Organohalide compounds are a giant group of halogen-substituted hydrocarbons produced in large quantities as solvents, plastics, pesticides, and chemical intermediates for industrial and agricultural uses (Stringer and Johnston, 2001; Jugder et al., 2016). The improper handling and disposal of harmful halogenated compounds resulted in their worldwide contamination in soil and water as well as bioaccumulation through food webs, posing threat to both human health and the environment (Stringer and Johnston, 2001; Zhou et al., 2004; Lu et al., 2017). Due to the side effects on biota, 69 out of the 126 EPA Priority Pollutants are organohalide compounds (United States Environmental Protection Agency, 2013). However, detection and monitoring of their environmental transport and fate using chromatography-based methods were limited due to the extremely wide variety of organohalide compounds (Stringer and Johnston, 2001).

Anoxic aquatic sediments became the major environmental sink for hydrophobic organohalide compounds, facilitating the growth of dehalogenating bacteria through organohalide-respiration (Smidt and de Vos, 2004; Zhou and Song, 2004; Rossi et al., 2012). In the organohalide-respiration process, anaerobic bacteria couple their growth with halogen-removal using acetate as a carbon source, H_2_ as an electron donor, and various organohalides as electron acceptors (Mohn and Tiedje, 1992; Holliger and Schumacher, 1994). Thus far, phylogenetically diverse bacterial groups have been identified to be able to remove halogens from organohalide compounds, including *Dehalococcoides*, *Dehalogenimonas*, *Dehalobium*, *Dehalobacter* and *Desulfitobacterium* (Smidt and de Vos, 2004; Zanaroli et al., 2015; Wang et al., 2016), which were normally originated from contaminated sites (Hendrickson et al., 2002; Taş et al., 2009; van der Zaan et al., 2010). Among them, *Dehalococcoides* are obligate organohalide-respiring bacteria that exclusively employ acetate as a carbon source, H_2_ as an electron donor and organohalides as electron acceptors to conserve energy for growth (Löffler et al., 2013). *Dehalococcoides* were identified to have the most diverse and extensive dehalogenation activities on organohalide compounds, including chloroethenes (Maymó-Gatell et al., 1997; He et al., 2003; Müller et al., 2004), chlorobenzenes (Adrian et al., 2000), polychlorinated biphenyls (PCBs) (Bedard et al., 2007; Wang et al., 2014), polybrominated diphenyl ethers (PBDEs) (He et al., 2006), chloroethanes and chlorophenols (Fennell et al., 2004; Lookman et al., 2004; Adrian et al., 2007; Wang and He, 2013a,b). Therefore, *Dehalococcoides* might be employed as a potential biomarker, complementing current chromatography-based methods, to test the presence of organohalide compounds.

In this study, we first screened *Dehalococcoides* in sludge and sediment samples collected from various anaerobic bioreactors for industrial wastewater treatment and contaminated black-odorous urban rivers. Further source-tracking together with laboratory cultivation confirmed which organohalide compounds supported the growth of *Dehalococcoides*. These results opened up opportunities employing *Dehalococcoides* as a biomarker to track unknown sources of organohalide compounds in wastewater and environmental samples.

## Materials and Methods

### Microbial Cultures Setup and Transfer

Sludge and sediment samples collected from bioreactors and black-odorous urban rivers were employed as inoculum for culture setup (**Table 1**). These samples were acquired directly by filling sterile 50 ml plastic Falcon tubes that were capped and transported to the laboratory at an ambient temperature. To control exposure of the samples to oxygen, Falcon tubes were sealed with Parafilm, and microcosm setup was performed in anaerobic chamber soon after their arrivals. For granular sludge, it was smashed into floc-form sludge before inoculation. Defined anaerobic mineral medium in 160 ml serum bottles for microbial cultivation was prepared as described (He et al., 2003; Wang and He, 2013a), which contains salts, trace elements and vitamins. L-cysteine and Na_2_S⋅9H_2_O (0.2 mM each) were added to the medium to achieved reduced conditions. The bottles were sealed with black butyl rubber septa and secured with aluminum crimp caps. The organohalide-fed cultures were transferred in 100 ml medium supplemented with 10 mM lactate, 10 mM 2-bromoethanesulphonate (BES, to inhibit methanogen growth), and 1 mM PCE or 10 ppm chloromethane. The control cultures without organohalide-amendment were transferred in the same mineral medium. Unless stated otherwise, cultures were incubated at 30°C in the dark without shaking. All the experiments were set up in duplicates.

**Table 1:** Sludge samples information which collected from anaerobic industrial wastewater treating bioreactors and environmental samples.

Sample No.	Sludge/sediments source	Sludge/sediments Form	Bioreactor type	*Dehalococcoides* occurrence	Dechlorination activity
1	Vitamin-C Industry	Granules	UASB	-	-
2	Petrochemical Industry	Granules	UASB	+	+
3	Brewery Industry	Granules	UASB	-	-
4	Paper mill Industry	Granules	UASB	-	-
5	Coke Industry	Flocs	Anaerobic digester	-	-
6	Acrylic textile Industry	Flocs	Anaerobic digester	-	-
7	Textile-dyeing Industry	Flocs	Anaerobic digester	-	-
8	WAS Anaerobic digestion Industry	Flocs	Anaerobic digester	-	-
9	Black-odorous River A	Flocs	N.A.	+	+
10	Black-odorous River B	Flocs	N.A.	+	+
11	Black-odorous River C	Flocs	N.A.	+	+


### Analytical Techniques

Headspace samples of chloroethenes (i.e., PCE, TCE, *cis*-DCE, *trans*-DCE, VC and ethane)and chloromethane were injected manually with a glass, gastight, luer lock syringe (Hamilton, Reno, NV, United States) into a gas chromatography (GC) 7890N equipped with a flame ionization detector (Agilent, Wilmington, DE, United States) and a GS-GasPro column (30 m × 0.32 mm; Agilent, Wilmington, DE, United States) as described (Wang and He, 2013b). The standards compounds (with analytical pure or above) were purchased from Sigma–Aldrich.

### Fluorescence *In Situ* Hybridization (FISH)

The FISH experiment was performed according to protocols described previously (Amann et al., 1995). Granular sludge samples were fixed in a 4% paraformaldehyde solution for 8 h at 4°C, and embedded in Optimal Cutting Temperature (O.C.T.) compound (Fisher Healthcare, Houston, TX, United States). Then the freezing granules were cut into 15 μm-thick sections with CM3050S cryostat (Leica, Germany). Hybridization was performed at 46°C for 4 h with oligonucleotide probes Dhe1259 (Yang and Zeyer, 2003), EUBmix and ARCH915 (Amann et al., 1995) targeting *Dehalococcoides*, bacteria and archaea, respectively. Dhe1259 and EUBmix/ARCH915 for dual-staining FISH were labeled with Cyanine 3 (Cy3) and Cy5, respectively. FISH-stained images were captured CLSM (Leica TCS-SP2, Germany).

### DNA Extraction, PCR, and Illumina Miseq Sequencing

Community gDNA was extracted using the FastDNA Spin Kit for Soil (MP Biomedicals, Carlsbad, CA, United States) according to the manufacturer’s instructions. The 16S rRNA gene was amplified with the U515F forward primer and U909R reverse primer as described (Narihiro et al., 2015). Illumina Miseq sequencing (Illumina, San Diego, CA, United States) service was provided by BGI (Shenzhen, China). The provided pair-end (2 × 300 nd) demultiplexed sequences were assembled and filtered using Mothur v.1.33 (Schloss et al., 2009). Quantitative Insights Into Microbial Ecology (QIIME, v1.8.0) was employed for the subsequent processing and downstream analysis (Caporaso et al., 2010).

### Data Deposition

Raw Illumina Miseq sequencing reads were deposited into NCBI Sequence Read Archive (SRA) with accession no. SRP112682.

## Results

### Screening of Obligate Organohalide-Respiring *Dehalococcoides* in Anaerobic Sludge and Sediment Samples

*Dehalococcoides* as an obligate dehalogenating bacterial group can only utilize organohalides as electron acceptors to support their growth (Löffler et al., 2013). In this study, sediment and sludge samples from black-odorous urban rivers and anaerobic bioreactors, respectively, were selected to screen the presence of *Dehalococcoides* (**Table 1**). PCR amplification with *Dehalococcoides* genus-specific primers, FpDHC1/RpDHC1377 (Hendrickson et al., 2002), showed the positive detection of *Dehalococcoides* in all urban river sediment samples, as well as in a granular sludge sample collected from a full-scale mesophilic UASB reactor treating petrochemical wastewater (**Table 1**). And the petrochemical wastewater contains organic compounds generated from terephthalic-acid industry, e.g., terephthalic-acid, benzoic acid, toluic acid, acetic acid and other intermediate compounds and byproducts (Lykidis et al., 2011).

**FIGURE 1:**
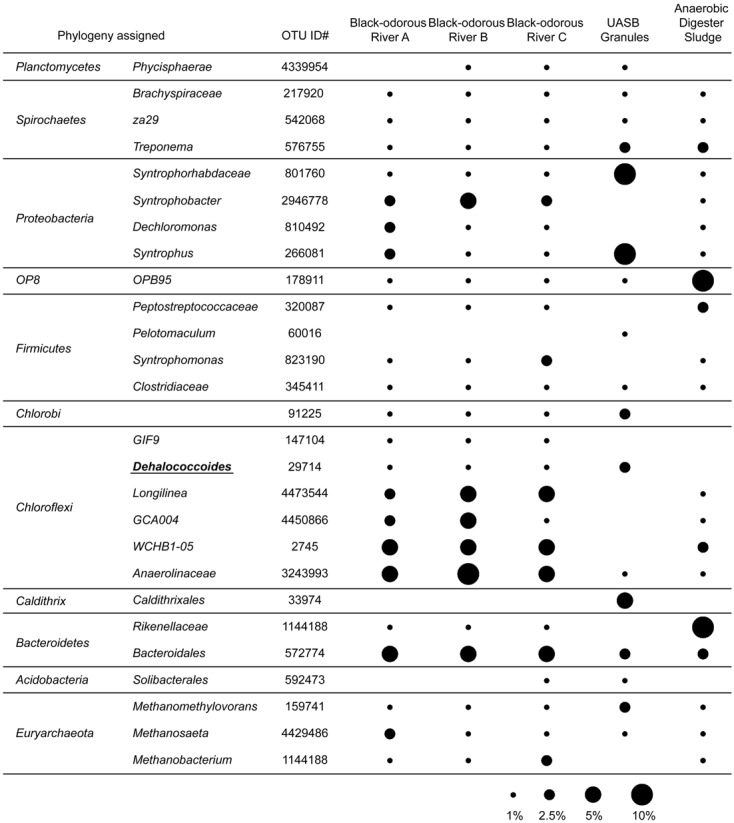
The relative abundance (RA) of dominant microbial populations in environmental samples. Only populations with RA > 0.5% in at least one samples were shown here.

To profile microbial communities of those *Dehalococcoides*-containing environmental samples, Miseq 16S rRNA gene sequencing was performed, showed the very different microbial community structure in samples between *Dehalococcoides*-containing granular sludge and urban river sediments (**Figure 1**). In granular sludge collected from the UASB reactor, acidogenic populations, *Syntrophorhabdus* (of *Syntrophorhabdaceae*) and *Syntrophus*, formed syntrophic interactions with methanogenic *Methanosaeta* and *Methanosarcinaceae* (**Figure 1**). Surprisingly, the obligate organohalide-respiring *Dehalococcoides* presented abundant in the full-scale UASB reactor, accounting for 1.83% of the total microbial community, comparable with the relative abundance of *Dehalococcoides* in enrichment cultures dechlorinating PCBs (Wang and He, 2013a) and PCE (Lee et al., 2015). The presence of abundant obligate organohalide-respiring *Dehalococcoides* implied that the TA-wastewater contained uncharacterized organohalide compound(s). In the UASB reactor, acetate and H_2_ generated from degradation of aromatic compounds in petrochemical wastewater by *Syntrophorhabdus*, *Syntrophus* and other syntrophs, together with low redox potential and the uncharacterized organohalide compounds, provide ideal growth niches for the fastidious *Dehalococcoides*. No other obligate dechlorinating bacteria, e.g., *Dehalogenimonas* and *Dehalobacter*, were found in the granular sludge sample. In a control sample collected from a lab-scale anaerobic sludge digester without organohalide amendment, no known dechlorinating bacteria can be detected (**Figure 1**). The highly similar microbial community structures of the three black-odorous river sediments, distinguish themselves from the community compositions of the granular sludge, especially the predominant lineages of *Chloroflexi* (i.e., *Longilinea*, *GCA004*, *WCHB1-05* and *Anaerolinaceae*) and *Proteobacteria* (i.e., *Syntrophobacter* and *Dechloromonas*) (Şimsir et al., 2017) (**Figure 1**). *Dehalococcoides* were shown the appearance in the microbial community, on which indicate the potential of organohalides’ contamination.

### Dechlorination Activities in *Dehalococcoides*-Containing Cultures

**FIGURE 2:**
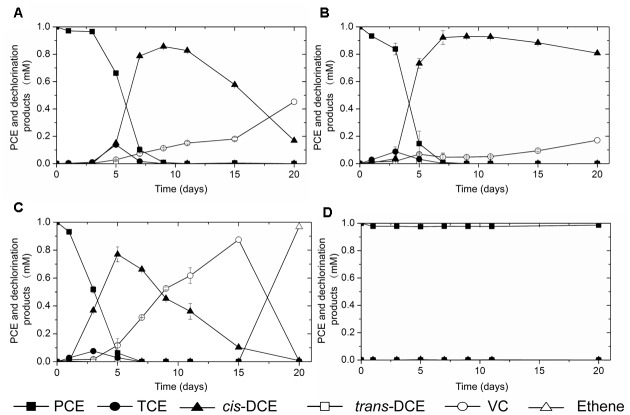
PCE-dechlorination activity observed in cultures inoculated with **(A)** sediment of black-odorous river A, **(B)** sediment of black-odorous river B, **(C)** sediment of black-odorous river C, **(D)** anaerobic digester sludge.

To further evaluate the dechlorination activities, perchloroethene (PCE) was spiked into microcosms established with those *Dehalococcoides*-containing sediment and sludge samples. After around 2 months’ incubation, PCE dechlorination activities were observed in all three microcosms with the river sediment inocula (data not shown). Subsequent consecutive culture transfers of the three microcosms generated three active cultures which reductively dechlorinate PCE into vinyl chloride (VC) or ethene (**Figure 2**). No dechlorination activity was observed in the control microcosm established with digester sludge (**Figure 2D**).

**FIGURE 3:**
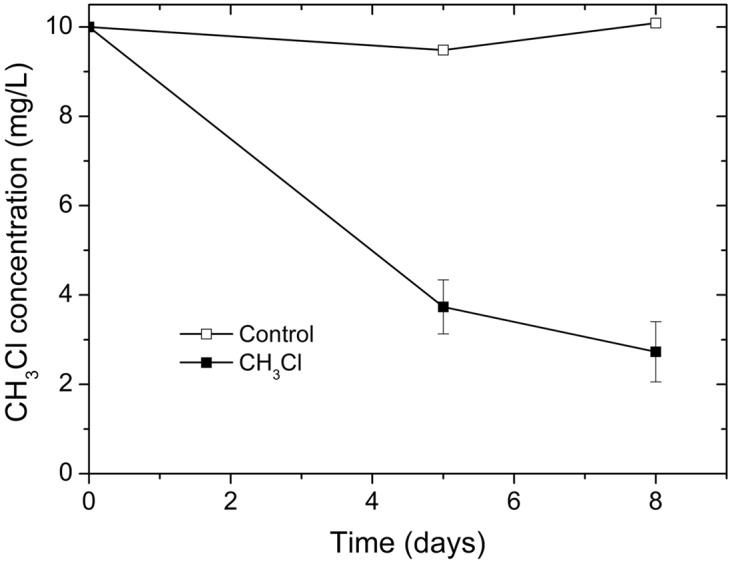
Dechlorination of chloromethane by UASB granules.

In contrast to PCE dechlorination in sediments of the three black-odorous urban rivers, microcosms inoculated with the *Dehalococcoides*-containing granular sludge showed negative PCE-dechlorination activity. To identify potential organohalides to support the growth of *Dehalococcoides* in the granular sludge, organohalide pollution in the petrochemical wastewater as influent of the UASB reactor was evaluated. The petrochemical wastewater was generated from a AMOCO process that oxidize *para*-xylene to terephthalic-acid, using a homogeneous catalyst of cobalt and manganese together with bromide as a promoter, in which bromomethane was generated as a byproduct (Tomás et al., 2013). Due to difficulties in obtaining bromomethane, dehalogenation activity test was performed with chloromethane as a homolog alternative to bromomethane. In chloromethane-fed culture, over 70% chloromethane was dechlorinated within 8 days (**Figure 3**). No obvious dechlorination activity was observed in abiotic control.

### *Dehalococcoides* in the Granular Sludge

**FIGURE 4:**
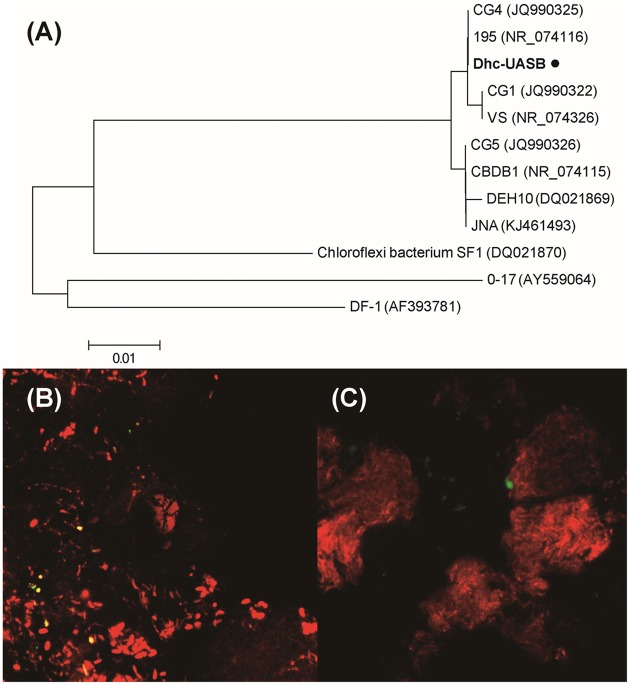
**(A)** Phylogenetic tree of *Dehalococcoides* identified in TA-degrading anaerobic granular sludge. Phylogenetic tree was calculated by neighbor-joining method using MEGA4 (Tamura et al., 2007). FISH analysis revealed the space distribution of **(B)** bacteria (red) and *Dehalococcoides* (yellow), and **(C)** archaea (red) and *Dehalococcoides* (green).

The partial 16S rRNA gene sequences (∼400 bp) generated from Miseq sequencing of V4–V5 hypervariable regions were unable to differentiate *Dehalococcoides* between Cornell and Victoria subgroups. Therefore, *Dehalococcoides* genus-specific primers (i.e., FpDHC1/RpDHC1377) were utilized to generate longer 16S rRNA gene sequences (∼1300 bp) to identify the *Dehalococcoides* in the anaerobic granular sludge. Phylogenetic analysis showed the close clustering of *Dehalococcoides* in TA-degrading granules with *D. mccartyi* 195 in Cornell subgroup (**Figure 4A**), sharing 99% 16S rRNA gene sequence similarity (2 bp difference over 1311 bp) with that of strain 195.

To provide insight into the spatial distribution of *Dehalococcoides* in the granular sludge, FISH was conducted with *Dehalococcoides*-specific, bacterial and archaeal oligonucleotide probes (Amann et al., 1995; Yang and Zeyer, 2003). FISH analysis showed the scattered distribution of *Dehalococcoides* inside granules, closely colonized with other bacteria (**Figure 4B**) but separated from archaea (**Figure 4C**). Degradation of aromatic compounds by fermentative bacteria is thermodynamically restricted and will become endergonic (ΔG > 0) as metabolic byproducts (e.g., acetate and H_2_) accumulate in the biosystem. Similar with methanogenic archaea, *Dehalococcoides* might form syntrophic interactions with aromatic compound degrading acidogens in the granular sludge: the degradation of aromatic compounds by *Syntrophorhabdus* and other syntrophs provide acetate as carbon source and H_2_ as electron donor for the halorespiration of *Dehalococcoides*; correspondingly, *Dehalococcoides* help maintain acetate and H_2_ at low concentration in the biosystem and ‘pull’ degradation of aromatic compounds toward completion through consuming metabolic byproducts generated by acidogenic bacteria. The close colonization of *Dehalococcoides* with syntrophic bacteria could facilitate the interspecies transfer of H_2_ (Mao et al., 2015).

## Discussion

Thus far, it remains challenging to detect organohalide compounds in wastewater and environmental samples based on chromatography methods due to their extremely wide variety, e.g., PCBs are a family of 209 structurally similar congeners (Chu and Hong, 2004; Elder et al., 2008). Bromomethane, similar with many other organohalide compounds produced as intermediate or byproducts in chemical synthesis processes, was a noteless synthesis byproduct in the petrochemical wastewater generated from terephthalic acid industry (Tomás et al., 2013). In this study, we reported the abundant presence of obligate organohalide-respiring *Dehalococcoides* in a full-scale UASB reactor for petrochemical wastewater treatment, and further cultivation experiments suggested the possible contamination of bromomethane in the petrochemical wastewater. Recent studies showed experimental evidences of biosynthesis of aromatic organohalides in nature, which might explain the detection of *Dehalococcoides* in the three black-odorous urban rivers (Agarwal et al., 2014; El Gamal et al., 2016; Şimsir et al., 2017). Also, *Dehalococcoides* was detected in various environmental samples contaminated with organohalides, including sludge/sediment collected from anaerobic digesters (Smith et al., 2015) and hyporheic zone of a wastewater treatment plant (WWTP)-impacted eutrophic river (Atashgahi et al., 2015). Therefore, *Dehalococcoides* might be a promising biomarker, complementing current chromatography-based methods, to test organohalide compounds in wastewater and environmental samples.

The UASB reactors provided ideal ecological niches for the growth of *Dehalococcoides* which further formed syntrophic interactions, as methanogens in syntrophic methanogenic communities (Stams and Plugge, 2009), with aromatic-compound degrading bacteria to overcome the thermodynamic limit through consuming acetate and H_2_. To our knowledge, this is the first report of the strictly organohalide-respiring *Dehalococcoides* present abundantly in a full-scale bioreactor for industrial wastewater treatment. In previous studies, *Dehalococcoides* was documented in various lab-scale bioreactors, including membrane biofilm reactors (Chung et al., 2008), UASB reactor (Hwu and Lu, 2008) and anaerobic biotrickling filter (Popat and Deshusses, 2009). The presence of *Dehalococcoides* in high abundance in both full- and lab-scale bioreactors showed the feasibility of removing toxic and persistent organohalides from various industrial wastewaters in anaerobic bioreactors through employing the microbial reductive dehalogenation process.

## Author Contributions

SW and DL conceived the idea. QL and LY performed the experiments and data analysis. SW, QY, TL, and ZH provided materials. QL, LY, and SW wrote the manuscript with inputs from all authors. All authors read and approved the final manuscript.

## Conflict of Interest Statement

The authors declare that the research was conducted in the absence of any commercial or financial relationships that could be construed as a potential conflict of interest. The reviewer JL declared a past co-authorship with one of the authors QL to the handling Editor.
